# Biofilm-Associated Amyloid Proteins Linked with the Progression of Neurodegenerative Diseases

**DOI:** 10.3390/ijms26062695

**Published:** 2025-03-17

**Authors:** Alka Ashok Singh, Fazlurrahman Khan, Minseok Song

**Affiliations:** 1 Department of Life Sciences, Yeungnam University, Gyeongsan 38541, Republic of Korea; alkasingh10f@yu.ac.kr; 2 Ocean and Fisheries Development International Cooperation Institute, Pukyong National University, Busan 48513, Republic of Korea; 3 International Graduate Program of Fisheries Science, Pukyong National University, Busan 48513, Republic of Korea

**Keywords:** biofilms, amyloid proteins, neurodegenerative diseases, pathogenesis, disease progression, molecular mechanisms

## Abstract

Biofilm-associated amyloid proteins have emerged as significant contributors to the progression of neurodegenerative diseases, representing a complex intersection of microorganisms and human health. The cross-beta sheet structure characteristic of amyloids produced by gut-colonizing bacteria remains intact, crucial for the resilience of biofilms. These amyloids exacerbate neurodegenerative disorders such as Alzheimer’s and Parkinson’s by cross-seeding human amyloidogenic proteins like amyloid-beta and α-synuclein, accelerating their misfolding and aggregation. Despite molecular chaperones and heat shock proteins maintaining protein homeostasis, bacterial amyloids can overwhelm them, worsening neuronal damage. Genetic variations in chaperone genes further influence amyloidogenesis and neurodegeneration. Persistent bacterial infections and inflammation compromise the blood-brain barrier, allowing inflammatory molecules and amyloids to enter the brain, perpetuating the cycle of neurodegeneration. The gut-brain axis underscores the impact of dysbiosis and gut microbiota on brain function, potentially contributing to neurodegeneration. The enhancement of biofilm resilience and antibiotic resistance by functional amyloid fibrils complicates the treatment landscape. The interplay among chaperone systems, microbial amyloids, and neurodegenerative diseases underscores the urgent need for advanced treatment strategies targeting these pathways to attenuate disease progression. Understanding the processes that relate biofilm-associated amyloids to the onset of neurological disorders is critical for diagnosing and developing novel treatment strategies.

## 1. Introduction

Bacterial infections pose serious hazards to human health, putting a huge strain on global healthcare systems. Notably, the World Health Organization (WHO) has categorized *Helicobacter pylori* as a class I carcinogen, emphasizing the severity of bacterial-induced diseases [1]. *Helicobacter pylori* causes gastric diseases such as gastritis. When bacteria enter the organelle, they create Listeriolysin O (LLO), which, in these acidic conditions, targets the membranes and causes the creation of pores, allowing bacteria to escape from the stressful and harmful environment of the phagolysosome [2]. Once in the cytoplasm, where the pH rises to 7, LLO toxic morphotypes are arrested form harmless amyloid aggregates, preventing the cytosolic membrane from being disrupted and potentially returning bacterial cells to the extracellular milieu. As a result, the several stages of toxin aggregation enable *L. monocytogenes* to efficiently adapt to the predominant environmental conditions. In this approach, amyloid characteristics play a critical role in modulating protein activity in a location-dependent manner [3]. Apart from serving a unique ecological niche, biofilms protect bacteria from their surroundings by increasing resistance to antimicrobials, protozoal grazers, UV radiation, desiccation, mechanical stress, and different host defenses [4,5,6]. Studies show posttranslational alterations contribute to the production and function of bacterial amyloids [7]. Bacterial presence in the gastrointestinal tract has adverse impacts on gastric epithelial cells and is a putative link to Alzheimer’s disease [8]. In cases of extreme oxidative stress, when damaged proteins accumulate, including crucial transcription factors necessary for Alkylhydroperoxide reductase (AhpC) production, *Helicobacter pylori* may reprogram AhpC as a molecular chaperone. This change enables the recovery of unfolded proteins, hence boosting *H. pylori* survival in the harsh environment of the human stomach. AhpC aids in protein folding and prevents aggregation, which is important for sustaining cellular function under stress, tying *H. pylori* to chaperone action and its ability to adapt to difficult situations [9,10]. Biofilms are surface-attached microbial colonies made up of many cell layers embedded in moist matrices [11]. During the maturation stage, bacterial cells produce extracellular polymeric substances (EPSs) such as polysaccharides, filamentous proteins, and extracellular DNA. These molecules combine to form an extracellular matrix (ECM), which envelops and supports the biofilm structure. This ECM is the distinguishing feature of bacterial biofilms and is important to their function [12]. Biofilms are clusters of single or multiple bacterial types encased inside a self-generated, three-dimensional ECM that support a wide range of gut-colonizing bacterial species. This matrix attaches strongly to both living and non-living surfaces [13]. It is widely acknowledged that bacteria are capable of producing amyloids, which are proteins with a conserved cross-beta sheet structure that resemble human amyloids in both structure and function [14]. The EPSs of biofilm-forming bacteria that include fibrils of amyloid protein are remarkably well-integrated and durable. Because of their special characteristics, these amyloid fibrils can interact with different elements of the matrix as well as pollutants in the environment [15]. Proteinaceous deposits of peptides that may be produced from larger precursor proteins, such as by proteolysis, are referred to as “amyloids”. All of these peptides have a stable secondary structure dominated by cross-β that promotes self-assembly into fibrils and insoluble oligomers. For an extensive period of time, human neurodegenerative illnesses have been primarily linked to these highly organized protein aggregates [16].

**Figure 1:**
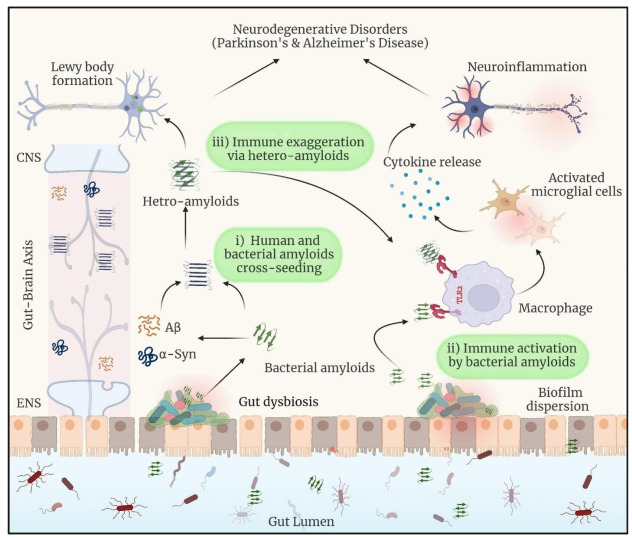
When dysbiosis releases bacterial amyloids into the gut, they can do the following: (**i**) start a cross-seeding process with human amyloidogenic proteins such as α-synuclein and Aβ; (**ii**) cause neuroinflammation by inducing the release of pro-inflammatory cytokines by the immune system; and (**iii**) form hetero-complexes with human amyloids, which may amplify the immune response. Reprinted with permission from [20], Copyright © 2024 Elsevier B.V.

One of the greatest problems facing modern medicine is the prevalence of age-related neurodegenerative disorders that include amyloid accumulation. A study reported that the microbiota from aged 3xTg AD mice exacerbated AD pathogenesis in young 3xTg mice, resulting in activation of the C/EBPβ/AEP signaling pathway in the brain. This contributes support to the idea that aged gut bacteria contribute to amyloid diseases in younger hosts [17]. More importantly, Aβ amyloidosis, plaque-localized microglia morphologies, and Aβ-associated degenerative alterations were all fully reversed in male mice treated with antibiotics (ABX) after receiving fecal microbiota transplantation (FMT) from transgenic (Tg) or wild-type (WT) male donors. Add this in after amyloids, which are structured scaffolds created by the assembly and aggregation of proteins, have been linked to neurodegenerative disorders such as PD and AD [18]. Changes in the gut microbiota actively contribute to the onset of neurological diseases. Enteric pathobionts have been shown to have a simpler version of cell surface proteins with amyloidogenic domains, known as facultative amyloids. Specifically, there is a relationship between the incidence of Parkinson’s disease (PD) and the presence of certain biofilm-associated protein (BAP) genes in the gut microbiome [19]. Amyloids, which are structured scaffolds created by the assembly and aggregation of proteins, have been linked to neurodegenerative disorders such as PD and Alzheimer’s disease (AD) [20,21]. Amyloids are formed whenever proteins assemble and aggregate together. Metal ions also play a complex role in the pathogenesis of AD and PD. Although many biological activities depend on these metals, a disruption in their equilibrium can result in the production of amyloid and neurotoxicity, which accelerates the process of neurodegeneration [20]. BAP, which is produced by *Staphylococcus aureus*, and Esp, which is produced by *Enterococcus faecalis*, are both examples of facultative amyloids [19,22]. Most neurodegenerative diseases, such as PD, AD, and type 2 diabetes, are characterized by the presence of inclusions and plaques composed of misfolded protein aggregates. In simple terms, interactions between homologous (same sequence) or heterologous (different) sequences produce these protein aggregates. Amyloid proteins exhibit cross-seeding, leading to a multi-component assembly, as demonstrated by several experimental findings [23]. According to the theory of “cross-seeding”, amyloid proteins from various origins may encourage one another’s aggregation. By facilitating the development and spread of amyloid plaques, microbial amyloids derived from biofilms have the potential to cross-seed the aggregation of human amyloidogenic proteins like α-synuclein or Aβ. This cross-seeding may facilitate the misfolding and build-up of amyloid proteins, which may exacerbate the pathogenic processes in neurodegenerative disorders [24,25] (Figure 1). Microbial proteins or metabolites may influence neurodegeneration by promoting amyloid production in human proteins or by increasing inflammatory responses to endogenous neuronal amyloids, but in a normal (healthy) gut, there is no compelling evidence that misfolded bacterial proteins cross-seed human amyloid and cause misfolding. However, exposure to bacterial amyloids can prime the immune system and increase the likelihood of misfolding in some circumstances, particularly in those with gut dysbiosis or pre-existing susceptibility to neurodegenerative disorders [26]. Bacterial amyloids may interact directly with human amyloids, causing misfolding and accelerating amyloid aggregation in disorders such as AD and PD. This cross-seeding method may aid in the pathological course of neurodegeneration. Furthermore, bacterial amyloids activate inflammatory responses, resulting in chronic neuroinflammation, which weakens the BBB and allows inflammatory chemicals and amyloids to enter the brain. This prolonged activation of inflammatory pathways worsens disease progression. Furthermore, functional bacterial amyloids are built to last and may serve as DNA carriers, triggering host immunological sensors and perhaps changing immune responses, resulting in the formation of autoantibodies. These combined actions cause a self-perpetuating loop of inflammation, amyloid buildup, and neuronal injury, eventually leading to neurodegeneration [14,27].

The interaction between molecular chaperones and amyloidogenic proteins is a crucial pathway in the development of neurodegenerative illnesses. Genetic differences in chaperone genes can also modulate the propensity for amyloidogenesis and the ensuing neurodegenerative disorders [28,29]. Biofilm-associated amyloids aid the advancement of neurodegenerative diseases because they cause chronic inflammation, encourage protein misfolding and aggregation, and strain the cellular chaperone mechanism. To unravel how neurological diseases progress through the involvement of BAPs and to devise novel therapeutic strategies for managing these conditions, this review explores the clinical significance of amyloid proteins in neurodegenerative diseases, the role of bacterial amyloid proteins, their evolutionary relationships, and potential therapeutic strategies.

## 2. Clinical Significance of Amyloid Proteins in Neurodegenerative Diseases

Amyloid proteins have significant clinical implications across various medical fields, particularly in the context of neurodegenerative diseases, systemic amyloidosis, and other pathological conditions. Here are the key clinical significances of amyloid proteins:

Amyloid-beta (Aβ) protein is critical in the etiology of AD. Sporadic AD is the leading cause of dementia, accounting for 50–56% of cases in autopsy and clinical investigations. The pathophysiology of AD is distinguished by two major features: (1) the presence of extracellular plaque deposits constituted of amyloid beta peptide, and (2) intracellular neurofibrillary tangles formed by the microtubule-binding protein Tau in a flame-shaped pattern. The creation and accumulation of amyloid beta peptides are critical to the development of AD and constitute the basis of the amyloid cascade hypothesis [30,31,32]. Aβ is generated through the cleavage of amyloid precursor protein (APP) by β- and γ-secretases [33].

Amyloid-β (Aβ) and tau accumulation have been linked to cognitive loss in PD. Research indicates that low Aβ42 levels in the cerebrospinal fluid (CSF) may predict cognitive impairment in PD. Recent research suggests that CSF Aβ is linked to postural instability and gait difficulties (PIGD), a recently identified cholinergic subtype of PD that may increase the likelihood of cognitive loss [34].

During Amyotrophic lateral sclerosis (ALS), each of the primary amyloid-like proteins in the nucleus mislocalizes to the cytoplasm. SOD1 loses its nuclear transport function and thus mislocalizes to the cytoplasm [35]. Native fused in sarcoma (FUS) is largely found in the nucleus; however, in ALS, it is mislocalized to the cytoplasm, notably dendritic stress granules [36]. TAR DNA-binding protein 43 (TDP-43) levels also lose shuttling capacity, with levels lowering in the nucleus and rising in the cytoplasm, serving as a reliable prediction of cell death. The mislocalization of proteins, like with most other amyloid-like NDs, appears to represent a constant pathological process in ALS [37,38,39].

Alzheimer’s disease (AD), named after the German psychiatrist Alois Alzheimer, is the most prevalent form of dementia. It progresses slowly and is characterized by the presence of neuritic plaques and neurofibrillary tangles resulting from the accumulation of Aβ peptide in the medial temporal lobe and neocortical structures [40]. Currently, roughly 50 million individuals worldwide are living with AD, and this figure is expected to triple every five years, reaching 152 million in 2050. AD has a worldwide effect on people, families, and economies, with yearly costs estimated to be about USD 1 trillion. While there is no cure for AD, there are drugs that may assist with symptoms [41,42]. AD is considered a complex condition since it is influenced by a number of risk factors, including age, genetics, head trauma, vascular disorders, infections, and environmental variables. The cause of AD pathological alterations, including Aβ accumulation, neurofibrillary tangles, and synapse loss, remains unclear. Several hypotheses have been proposed to explain the pathophysiology of AD. Possible causes include impaired cholinergic function and aberrant amyloid β-protein production and processing. Despite this, no definitive explanation for the pathophysiology of AD has yet been found [43,44].

**Figure 2:**
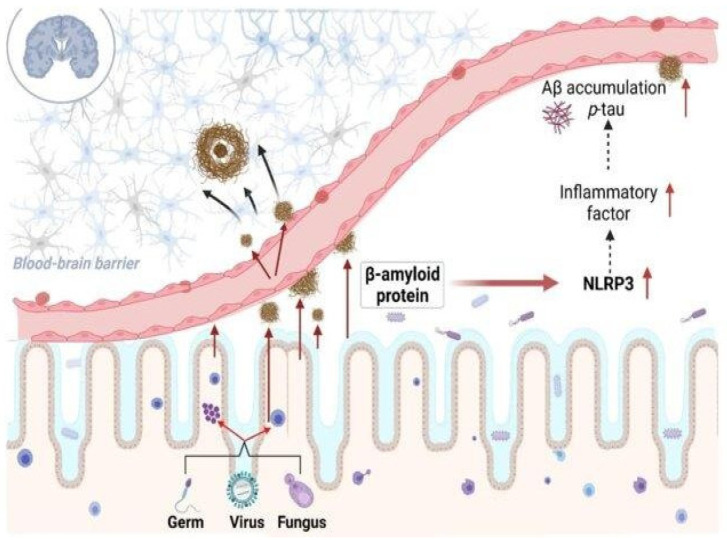
The role of bacteria-derived amyloid protein in inflammatory modulation in Alzheimer’s disease. Reprinted from [64], Copyright © 2023, License with author and Aging and Disease.

By 2025, it is anticipated that 6.5 million individuals in the United States will have AD, with forecasts increasing to 7.2 million [45]. Elevated levels of phosphorylated tau (p-tau) and amyloid beta (Aβ) in the brain precede cognitive deterioration in AD (Figure 2) [46]. Age-related cognitive decline and dementia are more common in older persons who are cognitively sound but have brain amyloidosis, which results in lower performance on long-term neurophysiological and cognitive examinations [47].

PD, the second most prevalent neurodegenerative ailment, causes symptoms such as tremors, rigidity, and bradykinesia [48]. It is characterized by the aggregation of α-synuclein in the brain [49]. Many PD patients retain self-sufficiency for the first decade following diagnosis, with younger patients and females having better clinical results [50]. According to the Global Burden of Disease Study, the prevalence, disability, and death rates for PD have all increased dramatically. The estimated worldwide prevalence of PD was 2.5 million in 1990, rising to 6.1 million in 2016, and expected to reach 8.4 million by 2019 [51]. PD has many causes, including hereditary and environmental factors. Pesticides are considered dangers, although physical activity and smoking may provide protection, albeit causation is complicated because to the protracted prodromal period [52]. Since the revelation of genetic linkages between α-synuclein and PD risk two decades ago, and the recognition of aggregated α-synuclein as a critical component in Lewy disease, α-synuclein has become the principal treatment target for PD and other synucleinopathies [53].

Patients with PD develop motor symptoms gradually, such as bradykinesia, resting tremor, and muscular stiffness. They may also develop non-motor symptoms, such as loss of smell (olfactory loss), mental problems, or cognitive impairments. These symptoms are caused by the gradual degradation and death of dopaminergic neurons in the substantia nigra. Together, these symptoms have a considerable influence on the patient’s quality of life [54]. Abnormal protein–protein interactions may exacerbate diseases, such as the dangerous self-assembly of α-synuclein in dopaminergic neurons in those with PD; therefore, small molecule regulation of α-synuclein aggregation is a promising therapeutic approach [55].

Amyotrophic lateral sclerosis (ALS) is among the most prevalent adult-onset motor neuron illnesses globally. It is defined by the gradual and selective loss of motor neurons in the motor cortex, brain stem, and spinal cord, resulting in muscle atrophy and fast-onset paralysis [56]. ALS, also known as Charcot disease or Lou Gehrig’s illness, is characterized by the breakdown of neuromuscular connections between upper and lower motor neurons, which leads to progressive muscle weakening, atrophy, and heightened tendon reflexes [57]. More than 120 genes have been linked to ALS [58,59]. It is believed that aberrant RNA processing causes the buildup of defective proteins, which contributes to the pathophysiology of ALS. Mutations in genes encoding RNA-binding proteins, such as TDP-43 and FUS, may cause familial ALS. Mutant variants of these proteins may accumulate in the cytoplasm rather than the nucleus, resulting in neurodegeneration. Furthermore, a non-coding hexanucleotide repeat expansion (C9ORF72) is the most prevalent genetic mutation associated with ALS, accounting for 45% of familial cases and 7% of sporadic instances, compared to 20% for the SOD1 gene [59,60]. The lifetime chance of having ALS is around one in 350 for men and one in 400 for women [61]. In 2020, there were an anticipated 121,028 cases of ALS in 22 European nations, with the United States reporting 800,000 [59]. By 2040, it is expected that there will be 380,000 additional cases of ALS globally [62]. The worldwide incidence varies from 1 to 2.6 per 100,000 persons, whereas the prevalence is 4 to 5 per 100,000 [62]. Incidence rates vary widely, ranging from 0.26 per 100,000 in Ecuador to 23.46 per 100,000 in Japan, whereas point prevalence varies from 1.57 per 100,000 in Iran to 11.80 per 100,000 in the USA [63].

## 3. Intricate Connections Between Microbial Biofilms-Associated Proteins and Neurological Disorders

The diet, which includes intakes of various macronutrients, including protein, accounts for around 20% of the microbial variance. Other exogenous and gut environmental factors also influence the composition and activity of colonic bacteria. While some bacterial species are specialists with a very small metabolic capacity, other species are often thought of as generalists and are able to metabolize a wide variety of substrates, including those produced from proteins and carbohydrates [65]. Consuming a diet rich in non-digestible carbohydrates—which the gut flora ferments in the colon—and limiting intake of proteins and fats are thought to be important ways to preserve gut health. By increasing the growth of particular members of the resident gut microbiota and promoting the generation of SCFAs, prebiotic supplements can lower pH and aid in pathogen exclusion [66].

The formation of amyloid-like fibrils by biofilm-associated proteins (BAPs) across a diverse range of microbial species has sparked widespread attention owing to its possible significance in neurological disorders. These proteins not only help maintain the structural integrity of biofilms, but they also have features similar to amyloid proteins seen in conditions like Alzheimer’s and Parkinson’s. Understanding the involvement of BAPs in amyloid production provides information not only about microbial biofilm dynamics but also about potential pathways driving neurodegenerative disease. The following sections demonstrate the intricate connections between microbial BAPs and neurological illnesses via the lens of BAP-induced amyloid-like fibril production. In addition, Table 1 highlights BAPs found in a variety of microorganisms. These proteins help create amyloid-like fibrils, which contribute significantly to the pathology of neurodegenerative disorders.

**Table 1:** List of biofilm-associated proteins (BAPs) from a diverse range of microbes that are involved in amyloid-like fibril formation and promote key pathological features of neurodegenerative diseases.

Name of BAPs	Microbes	Role in Microbial Life	Experimental Evidence in the Involvement of Neurodegenerative Diseases	References
Phenol-Soluble Modulins (PSMs)	*Staphylococcus aureus*	I. Colony spread, biofilm formation, and immune cell death, II. Production of pro-inflammatory cytokines in human keratinocytes is caused by pore creation, membrane rupture, and, ultimately, cell death.	I. PSMα3, with its monomeric and oligomeric forms, controls Aβ40 aggregation and plays a complex role in AD. II. The oligomer promotes Aβ40 aggregation, while the monomer prevents it, demonstrating a dual effect on the disease’s molecular pathogenesis.	[67,68]
Curli (CsgA and CsgB)	*Escherichia coli*	I. Curli formation, lipopolysaccharide (LPS) generation, and lysozyme inhibition. II. Adenosylcobalamin synthesis and oxidative stress response.	I. Curli exhibits cross-seeding and colocalization with α-syn in both *C. elegans* neurons and human neuroblastoma cells. II. Curli-induced α-syn aggregations inhibit mitochondrial gene expression, leading to energy failure in neurons. III. Curli may promote neuropathologies caused by many aggregation-prone proteins, including Aβ in Alzheimer’s disease, Huntingtin in Huntington’s disease, and SOD1 in amyotrophic lateral sclerosis.	[69]
FapC	*Pseudomonas genus*	I. FapC plays an important function in microbial life by encouraging biofilm development with its amyloidogenic characteristics.	I. FapC, especially its mutant version FapC ΔR1R2R3, inhibits the fibrillation of α-synuclein, which is a critical protein in Parkinson’s disease. II. The interaction is mediated by the production of disulfide bonds in FapC ΔR1R2R3. Bacterial amyloids may play a role in the onset and progression of Parkinson’s disease, as evidenced by the early presence of α-SN aggregates in GI tissues. III. FapC from gut bacteria like Pseudomonas may interact with α-SN in the GI tract, influencing its aggregation behavior.	[70]
TasA	*Bacillus subtilis*	I. *Bacillus subtilis* creates biofilms in which individual cells are kept together by an extracellular matrix.	I. TasA creates amyloid fibers. Amyloid fibers have been extensively researched in human neurodegenerative illnesses such as Alzheimer’s and Parkinson’s, as well as prion-induced spongiform encephalopathies. II. TasA restored wild-type biofilm morphology, demonstrating that the isolated protein preserved biological function.	[71]
Sup35	*S. cerevisiae*	I. Confers a translation termination malfunction and expression level-dependent toxicity in its amyloid form.	I. Injection of Sup35 fibrils into the striatum of wild-type mice caused α-synopathy and PD-like motor impairment. II. In vitro and in vivo, Sup35-seeded α-syn fibrils outperformed pure α-syn fibrils in terms of seeding activity and neurotoxicity. These data suggest that the yeast prion protein Sup35 causes α-synopathy in PD.	[72,73]
Bap	*Staphylococcus aureus*	I. In the N-terminal region of these proteins, there are brief sections with amyloidogenic potential. II. After proteolytic cleavage, the N-terminal region adopts a β-sheet-rich shape and polymerizes in acidic conditions, forming fibrillar structures that promote biofilm formation.	I. The presence of specific BAP genes in the gut microbiome is associated with the occurrence of Parkinson’s disease (PD). II. In cultured dopaminergic neurons and Caenorhabditis elegans models, we found that BAP-derived amyloids cause α-synuclein aggregation. III. Research findings indicate that BAP amyloids interfere with chaperone-mediated autophagy. Indeed, introducing BAP fibrils into wild-type mice brains promotes critical degenerative hallmarks of Parkinson’s disease.	[19,74,75]
enterococcal surface protein (Esp)	*Enterococcus faecalis*	I. The N-terminal region was not essential for biofilm development, but it dramatically fortified biofilms against mechanical or degradative disruption, hence enhancing Enterococcus retention within biofilms. II. Biofilm strengthening necessitated low pH, which caused Esp to unfold, aggregate, and form amyloid-like formations. The pH threshold for biofilm strengthening was determined by protein stability.	I. Research has been published showing a correlation between the function of Esp and a higher abundance of BAP genes in the context of neurological disorders, mainly Parkinson’s diseases. The binary logistic regression finding shows that there is a notably increased chance of the Esp gene being present in PD patients. II. Esp or Esp743, when produced from a plasmid in *E. faecalis* FA2-2, has been shown to result in enhanced biofilm development, as measured quantitatively by CV staining.	[19,75,76]

## 4. Biofilm Bacteria and Amyloid-Beta-like Proteins in Neurodegenerative Diseases

Oral biofilms, as well as related microbes such as *Porphyromonas gingivalis*, which are involved in disorders like periodontitis, have been connected to a variety of systemic human illnesses, including neurological diseases and cancers [77]. Clinical research has identified three biofilm-associated bacterial species known as the red complex, including *P. gingivalis*, *Tannerella forsythia*, and *Treponema denticola*, as being especially related to periodontal disease [78]. AD is characterized by amyloid-β (Aβ) buildup in the brain. Chronic infection with *P. gingivalis* stimulates CatB/NF-κB signaling pathways, causing Aβ buildup in inflammatory monocytes/macrophages and contributing to neurodegenerative disorders [79]. In vitro investigations have revealed that non-capsulated *P. gingivalis* may mimic AD-like changes, but a capsular strain (W83) of *P. gingivalis* reproduces important AD-related lesions in mice models. *P. gingivalis* generates proteolytic enzymes that degrade AβPP and tau, forming amyloid-β plaques and neurofibrillary tangles [80].

There are concerns that oral bacteria, particularly *P. gingivalis*, might enter the circulation and travel to the brain, causing inflammation, increasing the production of pro-inflammatory cytokines, and triggering infection, all of which could contribute to AD pathogenesis [81,82]. Other periodontal bacteria that form biofilms, such as *Actinomyces naeslundii*, *Prevotella intermedia*, *Fusobacterium nucleatum*, and *T. denticola*, have also been linked to the development and progression of AD, as evidenced by the presence of serum antibodies in AD patient [81].

BAPs have been shown to increase the aggregation and fibrillation of endogenous α-synuclein in PD models in *C. elegans* muscle and brain cells. BAP-amyloids are thought to cause α-synuclein pathology and PD in mice studies. Previous research has shown that injecting fibrillar α-synuclein into animal brains causes Lewy body pathology similar to PD [83,84]. Long-term studies on mice show deficits in fine motor ability after intrastriatal injection of rBapB-PFF. This suggests that BAP amyloids might worsen α-synuclein-related disorders and cause motor impairments. Because BAP genes are encoded in the microbiome’s auxiliary genomes, some bacterial strains may create BAP amyloids that have been linked to PD [19].

Amyloid-beta (Aβ) in the brain interacts with biofilm-associated amyloids to affect the pathophysiology of AD. These interactions may significantly worsen the development of extracellular Aβ plaques, a defining feature of AD. This is an in-depth account of how Aβ plaques impair neuronal activity and cause inflammatory reactions.

Understanding how the apolipoprotein E (APOE) genotype, the key genetic driver of AD risk, regulates cellular responses to Aβ plaques and neurofibrillary tangles (NFTs), as well as the microenvironment around these lesions, is critical. By tackling these problems, scientists may be able to provide an explanation for the observed differences in the rate of cognitive decline across AD patients [85,86].

CsgA, an amyloidogenic protein present in *Escherichia coli* biofilms in the gut, has been linked to PD in mice. This research implies that gut bacteria may impact or possibly cause human neurodegenerative illnesses such as Parkinson’s. Given that α-synuclein, the principal amyloidogenic protein in PD, has been demonstrated to promote amyloid formation in vitro, CsgA may have a comparable effect on human protein conformations and amyloid-related disorders. Other amyloidogenic proteins derived from gut bacteria and even food sources, such as persistent allergenic proteins, may potentially participate in these processes [87].

Animal studies show that exposure to curli-producing bacteria promotes both α-synuclein aggregation in the brain and α-synuclein deposition in the gut. The intestinal submucosal plexus of old Fischer 344 rats showed α-synuclein buildup [88]. Researchers used these rats to evaluate the effects of bacterial exposure (using an established oral delivery route) on α-synuclein deposition and aggregation in both the gut and the brain [89]. Rats treated with wild-type curli-producing bacteria showed higher α-synuclein deposition in stomach ganglion cells, hippocampal neurons (particularly the CA3 area), and striatum compared to rats subjected to curli-deficient mutant bacteria or vehicle control. Proteinase K was shown to be ineffective against brain deposits, suggesting aggregated α-synuclein. Curli-exposed rats showed considerably greater α-synuclein deposition in the gut, with an odds ratio of 2.5 (95% CI 4.4–239), suggesting a dramatically increased risk of higher gut scores than unexposed rats [90]. PD, AD, frontotemporal lobar degeneration, ALS, and other neurological illnesses are all linked to the production of pathogenic aggregated amyloid proteins with prion-like properties in neurons [83]. The prolonged metabolic activation of the brain’s default-mode network is thought to make the system susceptible to AD. Transgenic mouse studies show that neural activity correlates with interstitial fluid amyloid-β levels and plaque formation [83].

Amyloidogenic proteins, or proteins with the ability to generate amyloid fibrils, are an essential part of the biofilm matrix seen in many bacteria [91]. These fibrils support the biofilm’s structural stability and functional characteristics. Amyloid is a unique β-sheet-rich structure that can be acquired by a variety of proteins [92]. Amyloids are generally thought to be the result of protein misfolding and are frequently linked to neurodegenerative illnesses in humans, such as Alzheimer’s, Parkinson’s, and Huntington’s disorders. On the contrary, the amyloid fold is now understood to be an integral component of healthy cellular functioning [93].

The main component of curli fibrils, which comprise a significant portion of the *E. coli* biofilm and offer the bacterial community structural and chemical stability, is CsgA [94,95]. Unlike amyloid proteins associated with disease, such as Aβ and α-synuclein, which are mostly harmful because of uncontrolled fibrillation and oligomerization that can have cytotoxic effects, fibrillation-related proteins, like CsgA, are generated and organized into fibrils through a strict and regulated process [96,97]. A nucleator protein (CsgB), chaperones (CsgC and CsgE), and a specialized channel (CsgG) have all evolved in the *E. coli* cell. These proteins are all involved in the safe translocation of CsgA from the intracellular medium to the extracellular medium via the periplasm. CsgA is effectively converted into amyloid fibrils by attaching to the growing ends of fibrils that have already formed or by binding to the nucleator protein CsgB [95].

## 5. Biofilm Bacteria and α-Synuclein-like Protein Production in Neurodegenerative Diseases

**Figure 3:**
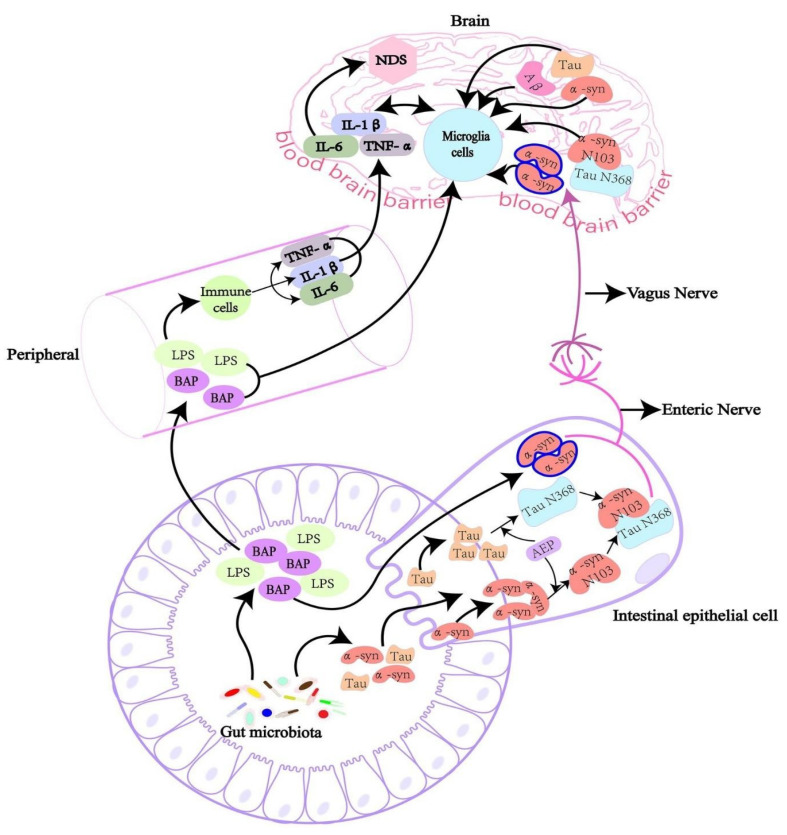
The intricate interactions between the gut microbiota, peripheral immune system, and brain, showing the mechanisms by which gut-derived substances might influence neuroinflammation and contribute to neurodegenerative disorders (NDS). Reprinted from [106], Copyright [2022/Frontiers] [Frontiers In Microbiology/Frontiers].

α-Synuclein aggregation has been linked to neurodegeneration in PD and other neurological disorders [98]. It is a neurodegenerative movement condition defined by dopaminergic neuron loss caused by α-synuclein oligomers [99]. The report suggests that cultured dopaminergic neurons and *C. elegans* models show that BAP-derived amyloids cause α-synuclein aggregation [19]. Feeding nematodes with *E. coli* VS39 strains producing amyloid-like fibers from BAPs results in increased α-synuclein aggregation and impaired motility, highlighting a potential mechanism by which gut microbiota can influence neurodegenerative disease development, according to research using the *C. elegans* NL5901model of PD (Figure 3) [19]. In mice that overexpress human amyloid α-synuclein, colonization with curli-producing *E. coli* causes α-synuclein disease in the gut and brain. Curli expression by *E. coli* exacerbates α-synuclein-induced behavioral problems, such as intestinal and motor deficits. Purified curli subunits increase α-synuclein aggregation, as shown in biochemical tests. The oral treatment of a gut-specific amyloid inhibitor lowers curli-mediated illness and behavioral impairments. This work shows that exposure to microbial amyloids in the gastrointestinal system may cause α-synuclein aggregation and illness symptoms in both the gut and brain [100]. The hyperphosphorylation of tau protein in the brain of an AD patient causes it to unbind from microtubules and destabilize the cytoskeleton, which ultimately contributes to the death of neurons through synapse disruption [101]. Hyperphosphorylated tau builds up inside cells and eventually outside of neurons to form neurofibrillary tangles [102]. The overproduction of Aβ and the hyperphosphorylation of tau appear to be mutually reinforcing diseases that lead to synaptic disruption and neurodegeneration [103]. Apart from tau and Aβ abnormalities, neuroinflammation has been identified as the third essential factor in the pathogenesis of AD. The central nervous system (CNS)’s major innate immune players, microglia, are able to identify and eliminate excessive Aβ accumulation [104]. On the other hand, a protracted activation of microglia sets off an inflammatory cascade that damages neurons. This may start the tau protein’s hyperphosphorylation, which encourages AD pathogenesis. However, as brains age, microglia lose their ability to scavenge, resulting in less Aβ deposits being removed, which facilitates AD pathogenesis. All of this shows that persistent AD can be exacerbated by neuroinflammation [105].

## 6. Biofilm Bacteria and TDP-43 Protein Production in Neurodegenerative Diseases

TAR DNA-binding protein 43 (TDP-43) is a highly conserved nuclear protein that regulates RNA/DNA binding and RNA processing. TDP-43 aggregates build in the central nervous system in a number of neurological illnesses, including ALS, frontotemporal dementia (FTD), and AD [107]. TDP-43 pathology is seen in 20% to 50% of AD patients, with a considerable majority (75%) having severe AD [108,109,110]. Another RNA-binding protein, FUS, has been linked to neurological diseases. In *C. elegans* models that express mutant SOD1 (G85R) linked with GFP, insoluble SOD1 aggregates accumulate in the perinuclear area of motor neurons, causing substantial locomotor deficits [111]. *C. elegans* models offer a solid foundation for systematically identifying bacterial components implicated in neurodegenerative disease (ND) pathogenesis. Recent research used *E. coli* single-gene knockout strains from the Keio library to scan the whole *E. coli* genome for genes that may be connected to neurodegeneration. Researchers used *C. elegans* models of PD to study the impact of missing certain *E. coli* genes on dopaminergic neuron loss and α-synuclein-induced locomotor deficits [112,113].

However, the function of oligomers in amyloid aggregation within condensates remains unclear. It was discovered that TDP-43 oligomers develop in the condensation and deposition routes and that they do so prior to the formation of solid aggregates within a condensed gel-like phase [114]. Additionally, it has been reported that α-synuclein oligomers form immediately after the monomeric protein phase-separated into a hydrogel. In these hydrogels, monomers, oligomers, and fibrils coexist, and α-synuclein oligomers and fibrillar α-synuclein are entrapped in a highly cytotoxic state rather than released [115]. This suggests that TDP-43-like oligomers could be produced by or influenced by bacterial biofilms, connecting bacterial infection and biofilm formation to TDP-43 aggregation in neurodegenerative disorders. Comprehending this link may offer fresh perspectives on the etiology of various illnesses and identify possible targets for treatment.

## 7. Mechanisms and Implications of Functional Amyloids in Biofilm-Forming Bacteria

**Figure 4:**
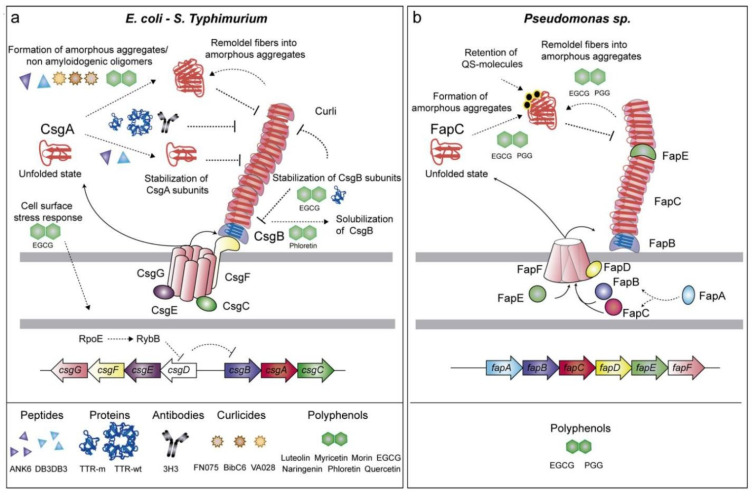
Interaction and regulation of amyloid fiber production in *E. coli* and *Pseudomonas* spp. (**a**) Curli fibers are built in *E. coli* and *S. Typhimurium* via the Csg pathway, with CsgA and CsgB producing amyloid fibers and CsgC, CsgE, and CsgF controlling aggregation. Polyphenols (EGCG and phloretin) prevent amyloid formation. (**b**) In *Pseudomonas* sp., FapC is the primary amyloidogenic protein, followed by FapB, FapD, and FapE, with QS molecules regulating aggregation. Polyphenols (EGCG and PGG) inhibit amyloid production by favoring amorphous aggregates. Reprinted from [124], Copyright © 2019 by the authors and Licensee MDPI, Basel, Switzerland.

In *Pseudomonas aeruginosa*, the development of functional amyloids is regulated by a single operon called fap, which contains six genes [116]. After removing a 24-residue signal peptide, the mature form of the main amyloid component, FapC, consists of 316 amino acid residues and three repeating sequences (R1, R2, R3) interspersed with two linker sections (L1, L2) [116,117]. The three repetitions are well conserved in pseudomonads and allied taxa, with an average residue conservation of 56% ± 25% across 65 strains [117]. As a result, current knowledge of FapC fibril production suggests that these repeat regions play an important role in amyloid polymerization, producing the core structure of mature fibrils [118]. The fap (functional amyloid in *Pseudomonas*) system is tightly controlled, similar to the Curli family [116,119]. FapC, a functional amyloid generated in *Pseudomonas* biofilms, induces α-synuclein fibrillation, whereas mutant FapC inhibits the process [70]. Infection of pulmonary microvascular endothelial cells with clinical *P. aeruginosa* strains that produce the bacterial amyloid FapC stimulates the formation of Aβ and tau proteins, which may spread to surrounding cells [120]. FapC is the primary component of Fap fibrils, and it contains a highly amyloidogenic area [121,122]. Its counterpart, FapB, functions as both a nucleator protein and a fibril component, with the absence of FapA changing the fibril composition from primarily FapC to predominantly FapB [116]. The translocation of FapC is a complicated interaction of components, including a porin-based membrane channel (FapF) and the water-soluble proteins FapD and FapE (Figure 4) [123].

In *Bacillus* species, TasA serves as the principal protein component in biofilms [125]. There are variations in TasA between phylogenetically distinct groups like the *Bacillus subtilis* and *Bacillus cereus* groups [126]. TasA is a stable, soluble, globular protein in its monomeric state, which transforms into filamentous structures during biofilm development. These filaments may have amyloid or non-amyloid properties and have been widely researched from functional, morphological, biochemical, and structural viewpoints. TasA native fibers have been directly shown to develop in vivo inside biofilm samples, and they can also be induced to form in vitro using pre-aggregated protein recovered from biofilms [71,127,128]. TasA filaments in these situations have a beta-sheet-rich structural arrangement while preserving a monomeric structure, which is controlled by donor strand complementation rather than the classic cross-beta architecture associated with normal amyloid proteins [129].

*B. subtilis* biofilms are mostly composed of exopolysaccharides and TasA protein. TasA may polymerize into fibers that mimic amyloid proteins in both biochemistry and morphology [130]. In *B. cereus*, as in *B. subtilis*, a particular chromosomal region is critical for biofilm formation [131]. This area contains one ortholog of sipW and two orthologs of *tasA* (*tasA* and *calY*). By reference to the genome sequence of the type strain *B. cereus* ATCC14579, all genes in this area were found in *B. cereus* CECT148. Notably, the genome of *B. cereus* ATCC14579 has a second putative ortholog (bc_4868), which shares 29% identity with *B. subtilis* TasA. This gene is anticipated to encode a putative protease and is found among other protease-encoding genes in the genome. TasA and, to a lesser degree, CalY have the innate capacity to polymerize and create fibers similar to TasA’s amyloid-like fibers in *B. subtilis*. Furthermore, the Congo Red-binding capacity seen in the pellicles of TasA-expressing *B. cereus* cells, as well as in *B. subtilis* cells supplemented with sipW-tasA and CalY or simply sipW-tasA, suggests that TasA fibers indeed display amyloid properties [132].

**Figure 5:**
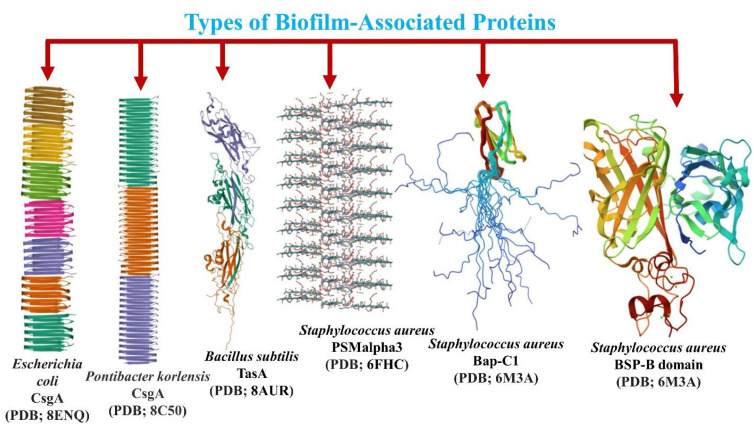
Three-dimensional structure of the biofilm-forming proteins from different species of bacteria with the properties of forming amyloid fibrils.

TasA, an amyloid protein, is important in Gram-positive organisms because it helps create biofilm matrices. TasA filaments perform two functions: They offer structural support to the biofilm’s ECM by connecting cells together, and when found in high quantities outside the cell as amyloids, they may release tiny aggregates to shield biofilm cells from prospective competitors. This conceptual framework needs thorough confirmation via more experiments [71]. TasA was first found as a spore-associated protein with antibacterial characteristics (Figure 5) [133], but it has now been shown to be the major protein in the ECM of *B. subtilis* biofilms [125].

## 8. Diversity of Biofilm-Associated Proteins and Their Amyloid Fibril Formation Properties

**Figure 6:**
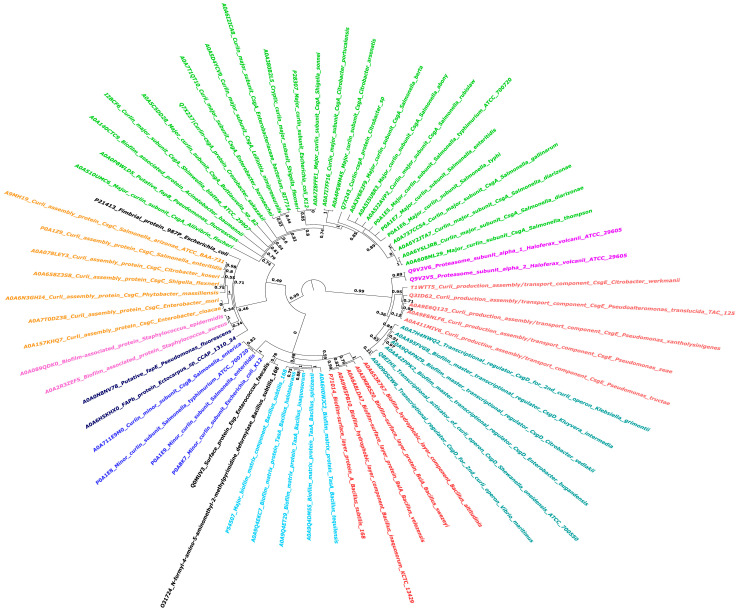
Phylogenetic analysis of Biofilm-Associated Proteins (BAPs) across various species of biofilm-forming microbes: This circular phylogenetic tree depicts the evolutionary relationships between different BAPs. To account for evolutionary changes, the tree was created employing FastTree, WAG, and gamma-distributed rate variation. Different colors depict BAPs clustering together, implying evolutionary conservation patterns. Bootstrap values appear at main nodes to indicate branch support.

The sequences of all biofilm-forming proteins from diverse bacterial species were retrieved from the National Center for Biotechnology Information (NCBI) database. The Fasttree package, using WAG (Whelan and Goldman) model for amino acid substitution, and Gamma-distributed rate variation to improve tree accuracy, was used to build the phylogenetic tree for these BAPs [134]. The phylogenetic tree presented depicts the evolutionary relationships between different bacterial species based on their primary curli subunit proteins, such as CsgA (Figure 6). Curli are functional amyloids produced by several bacterial species in the gut that aid in biofilm formation and host-cell attachment. The phylogenetic tree depicts the evolutionary relatedness of the BAPs of several Enterobacteriaceae species and related bacteria, emphasizing evolutionary divergence and distances. This tree contains multiple bacterial species and highlights the important curli subunit proteins, notably CsgA, which are essential for curli fiber production [135]. Each node on the tree reflects a common ancestor, and the branch lengths show the evolutionary distance between species. The tree contains many genera and species, all of which generate extracellular amyloid fibers containing curli proteins. These proteins are critical in biofilm formation, which is required for bacterial colonization and persistence in a variety of settings, including host organisms [136,137]. The curli-specific genes, known as the *csg* operon, code for both the major and minor curli fiber subunits, with *csgA* encoding the main curlin subunit [138]. Curli proteins found in several other species of biofilm-forming bacteria, as shown in the phylogenetic tree, may be connected to neurological disorders via immune system interactions. The tree emphasizes the evolutionary stability of curli proteins, demonstrating conservation between genera as well as close relatives within the same species. The Csg proteins from *E. coli*, known for their amyloidogenic capabilities, create amyloids with a unique cross-β-sheet structure [139]. These amyloids help maintain the biofilm’s protective and structural integrity. However, amyloids have been linked to various neurological disorders, including Huntington’s, Parkinson’s, and Alzheimer’s, in which misfolded protein aggregates build up in the brain, causing cell death and dysfunction [140]. TasA is the major protein component of biofilms in *Bacillus* species [141], with considerable variations across phylogenetically diverse taxa, such as *B. subtilis* and *B. cereus*. This resemblance may cause cross-reactivity, in which the immune system incorrectly attacks the body’s own amyloid proteins after exposure to bacterial curli proteins. These immune responses may aggravate neurological diseases. Molecular mimicry, which holds that curli fibers and pathogenic amyloids in neurological disorders exhibit structural characteristics, suggests a relationship between curli proteins and these diseases.

## 9. Conclusions and Future Perspectives

Amyloid proteins, such as Aβ and α-synuclein, are crucial in the progression of neurodegenerative diseases, including Alzheimer’s, Parkinson’s, and ALS. The creation, accumulation, and mislocalization of these proteins all contribute substantially to neurodegenerative diseases, highlighting the amyloid cascade hypothesis and its wide applicability to a variety of situations. Furthermore, some biofilm-forming bacteria, such as *P. gingivalis* and *E. coli*, have been linked to altered amyloid protein aggregation. These bacteria produce amyloid-like proteins, which may worsen neurological disorders, suggesting a complex interaction between microbial activity and neurodegenerative development. Such a connection underscores the need for a deeper understanding of the role of the microbiome in neurodegeneration and emphasizes the necessity of establishing therapeutic techniques that address both amyloid disease and microbial causes. Thus, investigating microbial–amyloid interactions and the potential for microbiome-based therapies could open new perspectives for preventing and treating diseases. Although animal models have supplied a wealth of information, they have had limited success translating these findings to human conditions. Research on the effects of bacterial infections and bacterial amyloids on neurodegenerative illnesses is still very appealing, even though no bacteria are currently known to produce a protein similar to TDP-43. Comprehending these interplays may yield novel perspectives on the mechanisms behind illnesses such as frontotemporal dementia and ALS. The phylogenetic analysis indicates that certain BAPs found in this pathogen are also identified in other bacterial pathogens, hinting at a potential involvement of this bacterial pathogen in neurodegenerative disease progression. Subsequent research should investigate how BAPs from these bacterial pathogens contribute to the advancement of neurodegenerative diseases. These types of studies offer new options for medicinal research, such as modulating microbiota to reduce amyloid pathology. Further study is needed to unravel the specific molecular and physiological pathways that connect amyloid proteins, microbial interactions, and neurodegenerative disease development. This improved knowledge may pave the way for novel diagnostic tools and tailored therapeutics aimed at halting amyloid accumulation, conserving neuronal integrity, and, eventually, improving outcomes for individuals suffering from these difficult illnesses. Future research will concentrate on discovering new biomarkers generated from microbial-amyloid interactions that might improve early detection and monitoring of neurodegenerative illnesses, thereby increasing diagnostic accuracy and treatment effectiveness.

## Data Availability

The data are contained within the article.

## Abbreviations

EPS	extracellular polymeric substances
ECM	extracellular matrix
PD	Parkinson’s disease
AD	Alzheimer’s disease
BAPs	bacterial-associated proteins
Aβ	amyloid beta
APP	amyloid precursor protein
PIGD	postural instability and gait difficulties
ALS	Amyotrophic lateral sclerosis
SOD1	superoxide dismutase 1
FUS	Fused in Sarcoma
p-tau	phosphorylated tau
TDP-43	TAR DNA-binding protein 43
*P. gingivalis*	*Porphyromonas gingivalis*
*T. denticola*	*Treponema denticola*
*E. coli*	*Escherichia coli*
*B. subtilis*	*Bacillus subtilis*
*B. cereus*	*Bacillus cereus*
APOE	apolipoprotein E
AβPP	amyloid-β protein precursor
PSM	phenol-soluble modulin
